# Type I Interferon Dependent hsa-miR-145-5p Downregulation Modulates MUC1 and TLR4 Overexpression in Salivary Glands From Sjögren’s Syndrome Patients

**DOI:** 10.3389/fimmu.2021.685837

**Published:** 2021-06-02

**Authors:** Daniela Jara, Patricia Carvajal, Isabel Castro, María-José Barrera, Sergio Aguilera, Sergio González, Claudio Molina, Marcela Hermoso, María-Julieta González

**Affiliations:** ^1^ Programa de Biología Celular y Molecular, Instituto de Ciencias Biomédicas, Facultad de Medicina, Universidad de Chile, Santiago, Chile; ^2^ Departamento de Tecnología Médica, Facultad de Medicina, Universidad de Chile, Santiago, Chile; ^3^ Facultad de Odontología, Universidad San Sebastián, Santiago, Chile; ^4^ Clínica INDISA, Santiago, Chile; ^5^ Escuela de Odontología, Facultad de Ciencias, Universidad Mayor, Santiago, Chile; ^6^ Programa de Inmunología, Instituto de Ciencias Biomédicas, Facultad de Medicina, Universidad de Chile, Santiago, Chile

**Keywords:** Sjögren’s syndrome, Type I interferons, hsa-miR-145-5p, Mucin 1, Toll-like receptor 4

## Abstract

Sjögren’s syndrome (SS) is an autoimmune disease that mainly affects salivary glands (SG) and is characterized by overactivation of the type I interferon (IFN) pathway. Type I IFNs can decrease the levels of hsa-miR-145-5p, a miRNA with anti-inflammatory roles that is downregulated in SG from SS-patients. Two relevant targets of hsa-miR-145-5p, mucin 1 (MUC1) and toll-like receptor 4 (TLR4) are overexpressed in SS-patients and contribute to SG inflammation and dysfunction. This study aimed to evaluate if hsa-miR-145-5p modulates MUC1 and TLR4 overexpression in SG from SS-patients in a type I IFN dependent manner. Labial SG (LSG) biopsies from 9 SS-patients and 6 controls were analyzed. We determined hsa-miR-145-5p levels by TaqMan assays and the mRNA levels of MUC1, TLR4, IFN-α, IFN-β, and IFN-stimulated genes (MX1, IFIT1, IFI44, and IFI44L) by real time-PCR. We also performed *in vitro* assays using type I IFNs and chemically synthesized hsa-miR-145-5p mimics and inhibitors. We validated the decreased hsa-miR-145-5p levels in LSG from SS-patients, which inversely correlated with the type I IFN score, mRNA levels of IFN-β, MUC1, TLR4, and clinical parameters of SS-patients (Ro/La autoantibodies and focus score). IFN-α or IFN-β stimulation downregulated hsa-miR-145-5p and increased MUC1 and TLR4 mRNA levels. Hsa-miR-145-5p overexpression decreased MUC1 and TLR4 mRNA levels, while transfection with a hsa-miR-145-5p inhibitor increased mRNA levels. Our findings show that type I IFNs decrease hsa-miR-145-5p expression leading to upregulation of MUC1 and TLR4. Together, this suggests that type I interferon-dependent hsa-miR-145-5p downregulation contributes to the perpetuation of inflammation in LSG from SS-patients.

## Introduction

Sjögren’s syndrome (SS) is a chronic autoimmune disease that primarily affects exocrine glands (mainly salivary and lachrymal), causing signs and symptoms of secretory dysfunction (1). SS-patients have increased systemic and glandular levels of pro-inflammatory cytokines, synthesized by inflammatory and epithelial cells (2–4). Accumulating evidence shows the involvement of interferons (IFNs) in both the initiation and progression of SS (5, 6).

IFNs are a family of cytokines originally defined by their anti-viral activity and are divided into three classes: type I, type II, and type III. The type I IFN class comprises 13 IFN-α subtypes, as well as IFN-β, IFN-ε, IFN-κ and IFN-ω. The type II IFN class comprises IFN-γ, while the type III IFN class consists of IFN-λ1, IFN-λ2 and IFN-λ3 (IL-29, IL-28A, and IL-28B, respectively) (6). Type I IFNs mainly signal through IFNα/β receptor (IFNAR), consisting of two subunits IFNAR1 and IFNAR2. Type I IFNs binds to IFNAR and transactivate the associated Janus protein kinases (JAKs) Tyk2 and Jak1, which are autophosphorylated. Activated Tyk2 and Jak1 then phosphorylate tyrosines on the intracellular receptor domains which recruit the Signal Transducer and Activator of Transcription (STAT) 1 and STAT2 heterodimers (6–8). The binding of interferon-regulatory factor 9 (IRF-9) to STAT1/STAT2 leads to the formation of a complex known as IFN-stimulated gene factor 3 (ISGF3). This complex induces the expression of IFN-stimulated genes (ISGs) by binding to IFN-stimulated response elements (ISRE) (6–8). Thus, type I IFN activation is commonly measured by evaluating the expression of ISGs, also called the “type I IFN signature” (6).

The ISG proteins have a wide variety of anti-viral/antineoplastic effector functions (9). However, type I IFNs also have several immunomodulatory roles, including isotype switching (10), induction of B cell-activating factor (BAFF) (11), and cytotoxicity mediated by T-cells and natural killers (12). Thus, tight regulation of type I IFN is required to avoid collateral tissue damage induced by excessive host defense responses (8).

Upregulated ISGs are present in labial salivary gland (LSG) biopsies and ocular epithelial cells (13) of SS patients using global gene expression profiling and real-time PCR (13–15). Among the most studied ISGs to determine type I IFN signature are Myxovirus (Influenza Virus) resistance 1, interferon-inducible (MX1); Interferon Induced Protein with Tetratricopeptide Repeats 1 (IFIT1); Interferon Induced Protein 44 (IFI44); and Interferon Induced Protein 44 Like (IFI44L) (16). In a microarray analysis using enriched epithelial cell fractions obtained from SS-patient LSG, MX1 and IFIT1 were upregulated (17). ISG overexpression was found in peripheral blood mononuclear cells (PBMCs), isolated monocytes, plasmacytoid dendritic cells, and B cells of SS-patients (13, 14, 18–21). Together, these observations indicate overactivation of the type I IFN pathway in SS. Systemic upregulation of type I IFN was associated with higher serum IgG, lower complement C3 levels, lower lymphocyte and neutrophil counts, and the presence of anti-Ro/SSA and anti-La/SSB autoantibodies (7, 22). Additionally, several studies show increased EULAR Sjögren’s syndrome disease activity index (ESSDAI) scores in type I IFN positive SS-patients (22–24). Local upregulation of type I IFN in the LSG of SS-patients was also associated with higher prevalence of abnormal findings on sialometry, leukopenia, hyperglobulinemia, high-titer antinuclear antibody, anti-SSA, and a high focus score of the LSG biopsy (25): emphasizing the key role of these cytokines in the pathogenesis of SS.

While the regulation and role of protein-coding genes involved in type I IFNs responses are well characterized, the involvement of non-coding microRNAs (miRNAs) is less studied (26). MiRNAs are single-stranded, non-coding small RNAs whose sizes range from 18 to 25 nucleotides. They play indispensable roles in regulating gene or protein expression by enhancing mRNA degradation or translational repression by binding to the 3’-untranslational region (3’-UTR) of target mRNAs (27, 28). Through their role in regulating gene expression, miRNAs participate in immune homeostasis and inflammatory responses, and their expression is altered in various autoimmune diseases, including SS (27). Interestingly, microRNAs themselves may be induced or repressed directly through type I IFN signaling (26). Type I IFNs inhibit miR-145 gene transcription, leading to decreased hsa-miR-145-5p levels (28), a miRNA with anti-inflammatory roles, and is downregulated in the LSG of SS-patients (29). The hsa-miR-145-5p inhibits IL-6 secretion in airway smooth muscle cells, and its absence leads to an induction of pro-inflammatory signals of the innate immune response (30, 31). Hsa-miR-145-5p levels decrease in T cells from systemic lupus erythematosus patients (32), as well as in skin biopsies and fibroblasts from systemic sclerosis patients (33), in the damaged renal vessels of patients with lupus nephritis (34, 35), and in PBMCs from patients with myasthenia gravis (36). Interestingly, two relevant hsa-miR-145-5p targets are overexpressed in LSG from SS-patients: mucin 1 (MUC1) (37, 38) and toll-like receptor 4 (TLR4) (39, 40), which have important roles in innate immunity, inflammation and glandular dysfunction.

MUC1 transcripts generate three types of isoforms: transmembrane proteins with or without variable numbers of tandem repeats (VNTR) and secreted proteins (37, 41–44). MUC1/SEC corresponds to secreted MUC1 isoform and does not contain the cytoplasmic and transmembrane domains (43, 45). Also this isoform is the only one that contains a unique 11 amino-acid peptide at the COOH terminus (43, 45). This sequence is known as immuno-enhancing peptide (IEP) and can enhance the immune response (46, 47). Interestingly, IEP modulates both the innate and adaptive immune responses (46, 48). Overexpression of cytokines may be induced by MUC1/SEC, mediated by IEP, and/or *via* the formation of complexes with MUC1/Y (44). The production of cytokines and modulation of the immune response could be triggered by the binding of MUC1/Y and MUC1/SEC (37, 49). MUC1/Y induces the transcription of pro-inflammatory cytokines *via* NF-kB (50). mRNA and protein levels of both isoforms are significantly increased in LSG from SS-patients (37). Moreover, MUC1 is overexpressed and accumulates in the endoplasmic reticulum (ER) of LSG from these patients. Pro-inflammatory cytokines induced aberrant MUC1 accumulation, suggesting that chronic inflammation alters the secretory process of MUC1, causing ER stress and could affect the quality of saliva in SS patients (38).

The Toll-like receptors (TLR) belong to a conserved family of type I transmembrane receptors, and each member recognizes distinct elements of bacteria, fungi, or viruses known as pathogen-associated molecular patterns (PAMPs) (51). TLR can also recognize damage-associated molecular patterns (DAMPs) such as histones, S100 proteins, heat shock proteins, and some extracellular proteins (52). TLR activation primes the adaptive immune system and initiates inflammatory responses by inducing proinflammatory cytokines, chemokines, co-stimulatory and adhesion molecules (53, 54). Furthermore, the development and progression of organ-specific autoimmune lesions in various experimental animal models suggest a role for TLR triggering in the pathogenesis of autoimmune disorders (55–57). This study specifically aimed to evaluate if hsa-miR-145-5p modulates the increased MUC1 and TLR4 expression in LSG from SS-patients in a type I IFN dependent manner.

## Materials and Methods

### Patients With SS and Controls

The study group included 9 patients diagnosed with primary SS, based on the 2016 American College of Rheumatology/European League against Rheumatism Classification Criteria (58). The control group included 6 subjects, who did not fulfill the primary SS classification criteria, did not suffer systemic diseases, and whose LSG biopsy analysis was normal or revealed mild diffuse chronic sialadenitis. **Table 1** summarizes the demographic, serological, and histological characteristics of SS-patient and control subjects. All individuals signed an informed consent according to the Declaration of Helsinki and the study was approved by the Ethics Committee of the Facultad de Medicina, Universidad de Chile (registration code CEISH 010-2016).

**Table 1:** Demographic and serological characteristics of SS-patients and control subjects.

	Control subjects	SS-patients
N° of individuals	6	9
Sex, N° female/N° male	4/2	8/1
Age, mean (range), years	40 (29-56)	37 (20-59)
Focus score†		
1	0	2
2	0	4
3	0	3
USWSF mL/15 min, mean (range)	3.8 (1.2-7.5)	2.0 (0-4.5)
Schirmer’s test ≤ 5 mm/5 min in at least one eye N° (%)	0 (0%)	4 (44%)
Ro antibodies N° (%)	0 (0%)	9 (100%)
La antibodies N° (%)	0 (0%)	5 (56%)
ANA antibodies N° (%)	1 (17%)	8 (100%)
RF N° (%)	0 (0%)	4 (44%)
ESSDAI, mean ± SD	–	13 ± 6
(range)		(5-19)

N°, number; %, percentage; SD, standard deviation; USWSF, Unstimulated whole salivary flow, ^†^Number of foci/4 mm^2^ of tissue. ANA, antinuclear; RF, Rheumatoid factor; ESSDAI, EULAR Sjögren syndrome disease activity index.

### Biopsies

The LSG biopsies were obtained as described by Daniels et al. (59). Collected samples were immediately frozen in liquid nitrogen.

### Taqman Assays

Total RNA extraction enriched in small RNAs was performed using the miRNeasy mini kit (QIAGEN Sciences, Maryland, USA). 40 ng of total RNA were reverse transcribed into cDNA using Taqman™ MicroRNA Reverse Transcription Kit (Applied Biosystems, CA, USA) and Taqman™ MicroRNA assays (Applied Biosystems, CA, USA) that include specific RT probes for hsa-miR-145-5p. To determine the expression levels of the miR-145-5p, Taqman™ Universal Master Mix II, no UNG (Applied Biosystems, CA, USA), and Taqman™ MicroRNA assays that include specific PCR probes for this miRNA were used. qPCR reactions for the hsa-miR-145-5p miRNA (20 μL reaction volume) were performed (at least in triplicate) using an MxPro 3000 termocycler (Stratagene). hsa-miR145-5p levels were normalized to those of the U48, and for calculating relative expression levels the efficiency-calibrated model was used (60).

### Real Time-PCR

Total RNA was extracted using the miRNeasy mini kit (QIAGEN Sciences, Maryland, USA). 1 µg of total RNA was reverse transcribed with oligo (dT), random primers, and the Superscript II enzyme (Invitrogen by Thermo Fisher Scientific, USA). Specific primers for the MUC1, TLR4, IFN-α, IFN-β, MX1, IFIT1, IFI44, IFI44L, and h18S genes were designed with the AmplifiX 1.4 software (**Supplementary Table 1**). For real-time PCR reactions, the 5X Hot FirePol EvaGreen qPCR Mix Plus (Solis BioDyne, Estonia) was used. The relative expression ratio of a target gene was compared to h18S. Relative quantification of target mRNAs was accomplished by comparative Ct, using the efficiency-calibrated model (60).

### Calculation of the Type I IFN Scores

The mean and standard deviation of each ISGs (MX1, IFIT1, IFI44, and IFI44L) obtained by real-time PCR in the control group LSGs were used to standardize the expression levels of each of these genes for each SS-patient, and IFN scores were calculated as described in previous studies (16, 22, 61, 62). Standardized expression levels were subsequently summed for each SS-patient to provide a type I IFN expression score, where i = each of the 4 IFN-inducible genes, Gene iSS= the gene expression level in each SS-patient, and Gene iCtr = the gene expression in controls:

$$\sum_{i=1}^{4}=\frac{Gene\;i_{SS}-mean\;\;Gene\;i_{Ctr}\;}{SD\;\left(Gene\;i_{Ctr}\right)}$$

### Type I IFN Stimulation and Functional Assays

Human submandibular gland (HSG) cells were cultured as previously described (40, 63) and incubated with or without 10 ng/mL human recombinant IFN-α (Biolegend, CA, USA) or IFN-β (R&D systems, MC, USA) in serum-free medium for 24 h and subsequently lysed to isolate RNA. For functional assays, HSG cells were transfected with 100 nM of specific hsa-miR-145-5p mimic and inhibitor (Applied Biosystems, CA, USA) at 24 h. Transient transfections were performed using HiPerFect transfection reagent (QIAGEN Sciences, Maryland, USA). Transfected HSG cells were lysed, and extraction of total RNA enriched in small RNAs was performed as described above.

### Protein Extraction and Western Blotting

HSG cells incubated with or without 10 ng/mL human recombinant IFN-α (Biolegend, CA, USA) or IFN-β (R&D systems, MC, USA) were homogenized using RIPA buffer and the Complete™ Protease Inhibitor Cocktail Mini Tablets (Roche, Mannheim, Germany). Proteins were quantified using the Bradford method (64) and separated according to their molecular weights by SDS-PAGE on 8% gels under reducing conditions. Separated proteins were transferred to nitrocellulose membranes (Bio-Rad Laboratories, Hercules, CA, USA) for 15 h at 4°C. Membranes were blocked for 1 h at room temperature (RT) in 5% skimmed-milk prepared in TBS-T buffer (10 mM Tris HCl [pH 7.5], 150 mM NaCl, 0.1% Tween 20). Blots were then separately incubated with primary antibodies against MUC1 or TLR4 (**Supplementary Table 2**) prepared in TBS-T buffer overnight at 4°C. After washes in TBS-T buffer, membranes were incubated with goat anti-mouse or anti-rabbit HRP-conjugated secondary antibodies (**Supplementary Table 2**) for 1 h at RT (Pierce^®^ by Thermo Scientific, IL, USA). Target proteins were detected by chemiluminescence (Pierce, IL, USA). Protein bands were quantified by densitometry. Protein levels were normalized to β-actin.

### Statistical Analysis

Mean values in SS-patients and controls groups or between different culture cell conditions were compared using the Mann-Whitney test. Spearman rank correlation analysis was also performed with P values less than 0.05 considered significant.

## Results

### hsa-miR-145-5p Is Downregulated in LSG From SS-Patients and Inversely Correlates With IFN-β and Type I IFN Score

**Figure 1:**
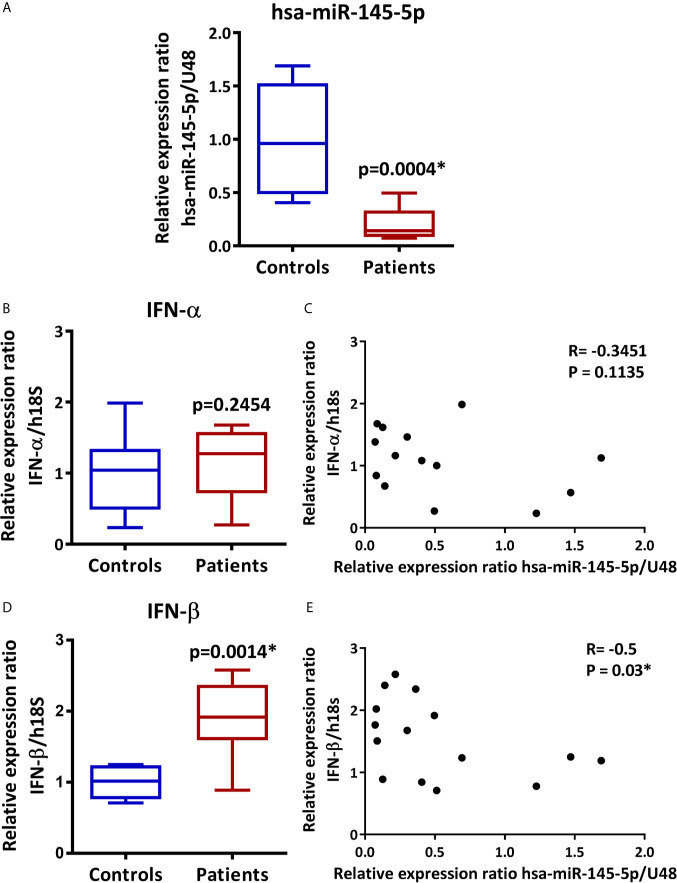
hsa-miR-145-5p is downregulated in LSG from SS-patients and inversely correlates with IFN-β mRNA levels. **(A)** hsa-miR-145-5p levels in SS-patients (n=9) and control subjects (n=6). U48 snRNA was used as a housekeeping. **(B)** IFN-α transcript levels in SS-patients and control subjects. h18S was used as a housekeeping gene. **(C)** Spearman correlation between hsa-miR-145-5p and IFN-α mRNA levels in SS-patients and controls. **(D)** IFN-β transcript levels in SS-patients and control subjects. h18S was used as a housekeeping gene. **(E)** Spearman correlation between hsa-miR-145-5p and IFN-β mRNA levels in SS-patients and controls. Data are representative of at least three independent measurements. (*) p-value ≤ 0.05 was considered significant.

Using TaqMan miRNA assays in LSG from 9 SS-patients and 6 controls, we validated hsa-miR-145-5p level. We observed decreased expression in SS-patients compared to controls (p=0.0004) (**Figure 1A**). Then, we assessed IFN-α and IFN-β transcript levels and their association with hsa-miR-145-5p. IFN-α transcript levels were near the limit of detection and no significant differences were found between groups (p=0.25) (**Figure 1B**), while IFNβ transcript levels were significantly increased in LSG from SS-patients compared to controls (p=0.0014) (**Figure 1D**). Spearman’s analysis showed a negative correlation between hsa-miR-145-5p and IFN-β (**Figure 1E**). However, there was no correlation between miR-145-5p and IFN-α (**Figure 1C**).

**Figure 2:**
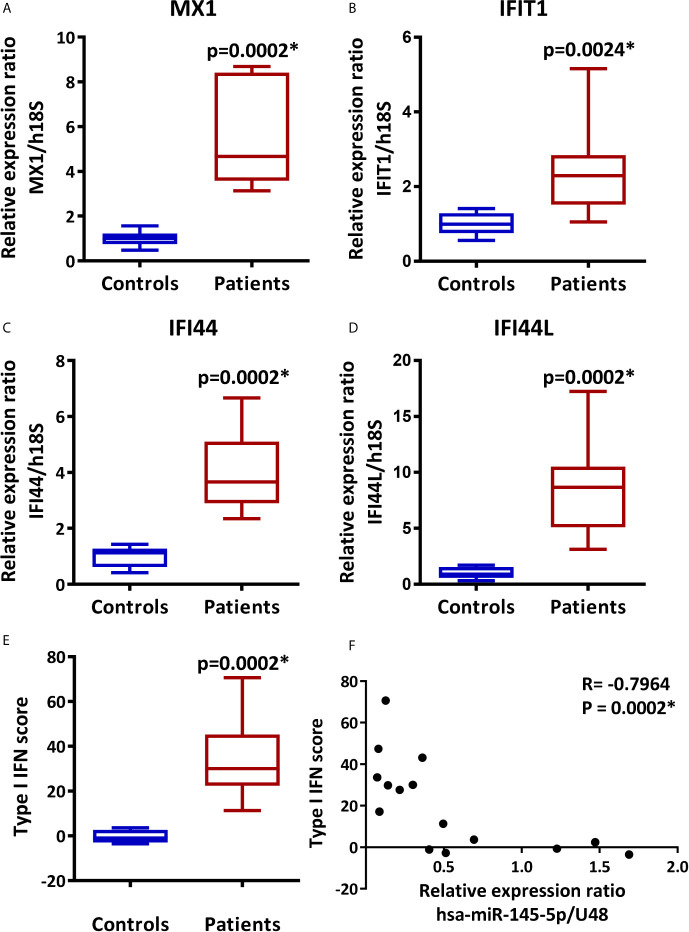
Overexpression of MX1, IFIT1, IFI44, and IFI44L and increased type I IFN score in LSG from SS-patients. Relative expression ratios of MX1 **(A)**, IFIT1 **(B)**, IFI44 **(C)** and IFI44L **(D)** in 9 SS-patients and 6 controls. h18S was used as a housekeeping gene. **(E)** Type I IFN score was calculated by summing the MX1, IFIT1, IFI44, and IFI44L standarized expression levels. **(F)** Spearman correlation between hsa-miR-145-5p levels and type I IFN score in SS-patients and controls. Data are representative of at least three independent measurements. (*) p-value ≤ 0.05 was considered significant.

The assessment of type I IFN score by real-time PCR analysis of multiple genes preferentially induced by type I IFNs or ISGs is a useful technique to determine the activation of the type I IFN pathway or type I IFN signature (16, 62). Therefore, the relative expression of MX1, IFIT1, IFI44, and IFI44L and the type I IFN score in LSG from SS-patients and controls were determined. As shown in **Figure 2**, mRNA levels of MX1 (p=0.0002), IFIT1 (p=0.0024), IFI44 (p=0.0002), and IFI44L (p=0.0002), were significantly higher in LSG from SS-patients compared to controls (**Figures 2A–D**). We calculated the type I IFN score and the results showed that the mean IFN score in SS patients was 34.5, with a range of 11.2 to 70.6, and in controls, the mean IFN score was -0.3, with a range of -3.5 to 3.6. Thus, the type I IFN score was significantly higher in SS-patients (p=0.0002) (**Figure 2E**), and there was a negative correlation between hsa-miR-145-5p levels and type I IFN score (p=0.0002) (**Figure 2F**).

### MUC1 and TLR4 Are Overexpressed in LSG From SS-Patients and Inversely Correlate With hsa-miR-145-5p

**Figure 3:**
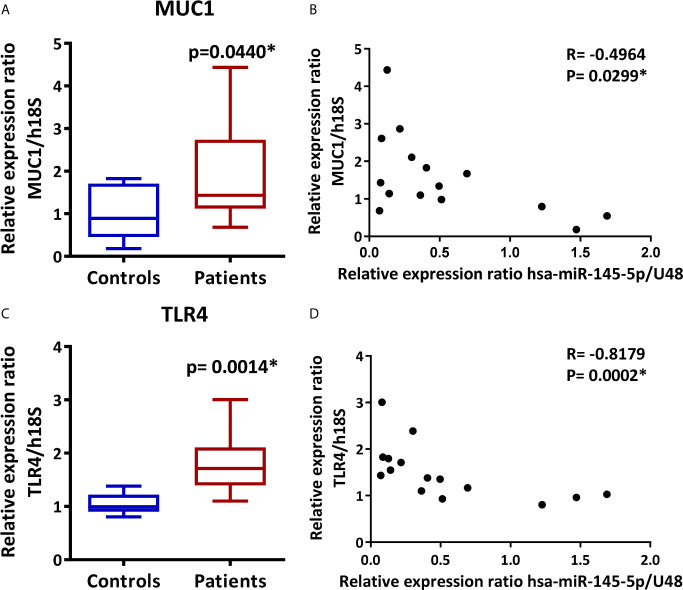
MUC1 and TLR4 are overexpressed in LSG from SS-patients and inversely correlate with hsa-miR-145-5p. **(A)** MUC1 transcript levels in SS-patients and control subjects. h18S was used as a housekeeping gene. **(B)** Spearman correlation between hsa-miR-145-5p and MUC1 mRNA levels in SS-patients and controls. **(C)** TLR4 transcript levels in SS-patients and control subjects. h18S was used as a housekeeping gene. **(D)** Spearman correlation between hsa-miR-145-5p and TLR4 mRNA levels in SS-patients and controls. Data are representative of at least three independent measurements. (*) p-value ≤ 0.05 was considered significant.

We measured MUC1 and TLR4 transcript levels by real-time PCR in LSG samples and evaluated their association with hsa-miR-145-5p. Results showed an increase in MUC1 and TLR4 transcripts levels in SS-patients (p=0.044 and 0.0014, respectively) (**Figures 3A, C**). Spearman’s analysis showed a negative correlation between hsa-miR-145-5p and MUC1 transcript levels (**Figure 3B**) and between hsa-miR-145-5p and TLR4 (**Figure 3D**) transcript levels.

### hsa-miR-145-5p Levels Are Inversely Correlated With Glandular Inflammation

According to its anti-inflammatory role, we found a negative correlation between hsa-miR-145-5p levels and clinical parameters of SS-patients: Ro autoantibodies (r= -0.82, p<0.001); La autoantibodies (r= - 0.59, p= 0.021), and focus score (r= -0.81, p<0.001) (**Table 2**). Additionally, we observed a positive correlation between these clinical parameters and mRNA levels of TLR4, ISGs, and type I IFN score (**Table 2**). ISGs negatively correlated with hsa-miR-145-5p levels, while ISGs and mRNA levels positively correlated with IFN-β and TLR4 (**Table 3**).

**Table 2:** Spearman’s rank correlation coefficients between hsa-miR-145-5p levels, ISGs mRNA levels and clinical parameters of SS-patients and control subjects.

Parameters	Spearman’s rho	p
Ro - miR-145	-0.819	<0.001
Ro - mRNA IFN-β	0.756	0.001
Ro - mRNA IFI44	0.850	<0.001
Ro - mRNA IFI44L	0.850	<0.001
Ro - mRNA MX1	0.850	<0.001
Ro - mRNA IFIT1	0.724	0.002
Ro - Type I IFN score	0.850	<0.001
Ro - mRNA TLR4	0.756	0.001
La - miR-145	-0.589	0.021
La - mRNA IFI44	0.655	0.008
La - mRNA IFI44L	0.589	0.021
La - mRNA IFIT1	0.556	0.031
La - Type I IFN score	0.589	0.021
Ana -mRNA IFN-β	0.756	0.001
Ana - mRNA IFI44	0.850	<0.001
Ana - mRNA IFI44L	0.850	<0.001
Ana - mRNA MX1	0.850	<0.001
Ana - mRNA IFIT1	0.724	0.002
Ana - Type I IFN score	0.850	<0.001
Ana - mRNA TLR4	0.756	0.001
Focus score - miR-145	-0.807	<0.001
Focus score - mRNA IFI44	0.849	<0.001
Focus score - mRNA IFI44L	0.737	0.002
Focus score - mRNA MX1	0.642	0.010
Focus score- mRNA IFIT1	0.724	0.002
Focus score - Type I IFN score	0.752	0.001
Focus score - mRNA TLR4	0.598	0.019

**Table 3:** Spearman’s rank correlation coefficients between hsa-miR-145-5p levels and mRNA levels of each analyzed gene in LSG from SS-patients and control subjects.

Molecules	Spearman’s rho	p
miR-145 - mRNA IFI44	-0.821	<0.001
miR-145 - mRNA IFI44L	-0.836	<0.001
miR-145 - mRNA MX1	-0.739	0.002
miR-145 - mRNA IFIT1	-0.682	0.007
mRNA IFN-α - mRNA MUC1	0.578	0.033
mRNA IFN-β - mRNA IFI44	0.632	0.014
mRNA IFN-β - mRNA IFI44L	0.579	0.026
mRNA IFN-β - mRNA MX1	0.704	0.005
mRNA IFN-β - mRNA IFIT1	0.607	0.019
mRNA IFN-β - Type I IFN score	0.596	0.021
mRNA IFI44 - mRNA IFI44L	0.914	<0.001
mRNA IFI44 - mRNA MX1	0.875	<0.001
mRNA IFI44 - mRNA IFIT1	0.918	<0.001
mRNA IFI44 - Type I IFN score	0.936	<0.001
mRNA IFI44 - mRNA TLR4	0.618	0.016
mRNA IFI44L - mRNA MX1	0.907	<0.001
mRNA IFI44L - mRNA IFIT1	0.861	<0.001
mRNA IFI44L - Type I IFN score	0.982	<0.001
mRNA IFI44L - mRNA TLR4	0.775	0.001
mRNA MX1 - mRNA IFIT1	0.796	<0.001
mRNA MX1 - Type I IFN score	0.946	<0.001
mRNA MX1 - mRNA TLR4	0.664	0.009
mRNA IFIT1 - Type I IFN score	0.879	<0.001
mRNA IFIT1 - mRNA TLR4	0.600	0.020
Type I IFN score - mRNA TLR4	0.700	0.005
mRNA MUC1 - mRNA TLR4	0.721	0.003

### Type I IFN Stimulation Downregulates hsa-miR-145-5p and Induces MUC1 and TLR4 Overexpression in HSG Cells

**Figure 4:**
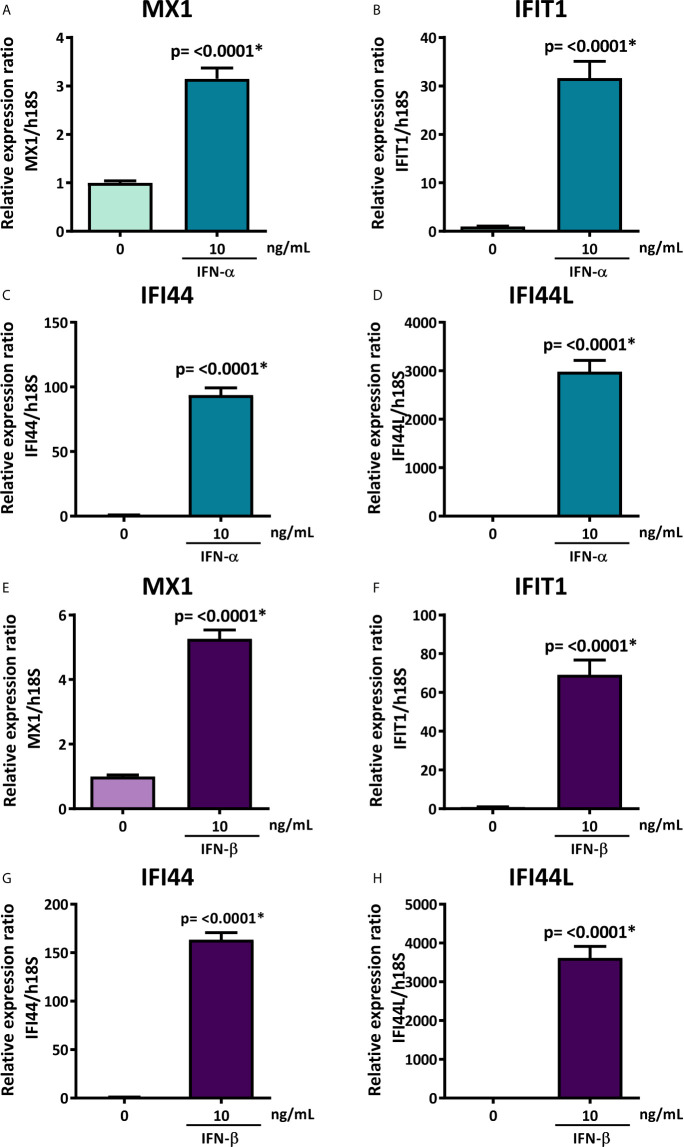
Overexpression of MX1, IFIT1, IFI44, and IFI44L in IFN-α or IFN-β-stimulated HSG cells. Relative expression ratios of MX1 **(A)**, IFIT1 **(B)**, IFI44 **(C)** and IFI44L **(D)** in HSG cells stimulated with or without 10 ng/mL human recombinant IFN-α for 24 h. h18S was used as a housekeeping gene. Relative expression ratios of MX1 **(E)**, IFIT1 **(F)**, IFI44 **(G)** and IFI44L **(H)** in HSG cells stimulated with or without 10 ng/mL human recombinant IFN-β for 24 h. h18S was used as a housekeeping gene. Data are representative of at least three independent experiments. (*) p-value ≤ 0.05 was considered significant.

**Figure 5:**
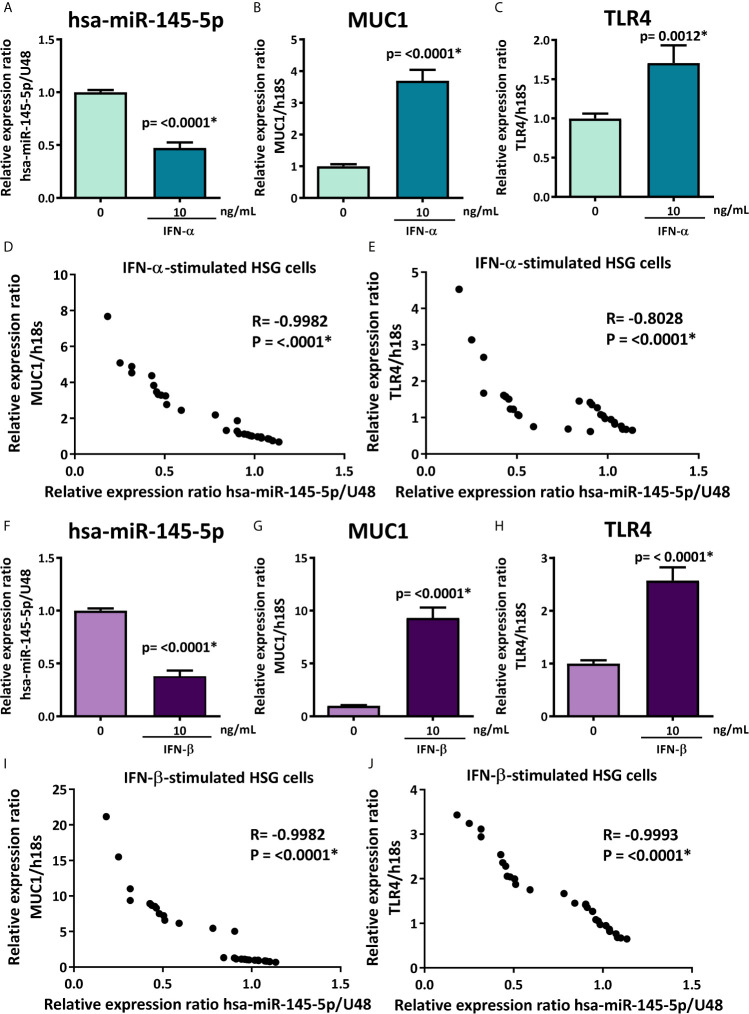
hsa-miR-145-5p is downregulated and MUC1 and TLR4 are overexpressed in type I IFNs-stimulated HSG cells. **(A)** hsa-miR-145-5p levels in HSG cells stimulated with or without 10 ng/mL human recombinant IFN-α for 24 h. U48 was used as a housekeeping gene. Transcript levels of MUC1 **(B)** and TLR4 **(C)** in HSG cells stimulated with IFN-α. Spearman correlation between hsa-miR-145-5p and MUC1 **(D)** or TLR4 **(E)** transcript levels in IFN-α stimulated HSG cells. **(F)** hsa-miR-145-5p levels in HSG cells stimulated with or without 10 ng/mL human recombinant IFN-β for 24 h. U48 was used as a housekeeping gene. Transcript levels of MUC1 **(G)** and TLR4 **(H)** in HSG cells stimulated with IFN-β. Spearman correlation between hsa-miR-145-5p and MUC1 **(I)** or TLR4 **(J)** transcript levels in IFN-β stimulated HSG cells. Data are representative of at least three independent experiments. (*) p-value ≤ 0.05 was considered significant.

**Figure 6:**
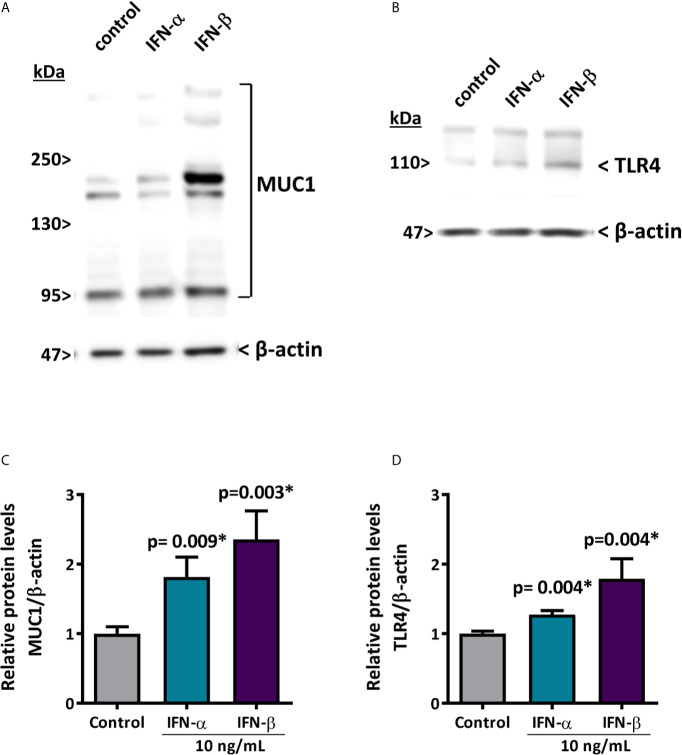
IFN-α and IFN-β increase MUC1 and TLR4 protein levels in HSG cells. **(A, C)** representative MUC1 western blot (90-300 kDa) in HSG cells stimulated with 10 ng/mL IFN-α or IFN-β for 24 h. Relative protein levels were normalized to the control condition. β-actin was used as a loading control. **(B, D)** representative TLR4 western blot (95 kDa) in HSG cells stimulated with 10 ng/mL IFN-α or IFN-β for 24 h. Relative protein levels were normalized to the control condition. β-actin was used as a loading control. Data are representative of at least three independent experiments. (*) p-value ≤ 0.05 was considered significant.

To evaluate if the decreased levels of hsa-miR-145-5p observed in SS-patients are associated with type I IFNs and the activation of this specific pathway, we assessed the effect of IFN-α or IFN-β stimulation on hsa-miR-145-5p levels and its targets MUC1 and TLR4 in HSG cells. First, to assess type I IFN pathway activation, we measured ISG expression in HSG cells stimulated with IFN-α or IFN-β. Stimulation with 10 ng/mL IFN-α induced an increase of MX1 (p<0.0001) (**Figure 4A**), IFIT1 (p<0.0001) (**Figure 4B**), IFI44 (p<0.0001) (**Figure 4C**), and IFI44L (p<0.0001) (**Figure 4D**) mRNA levels. In addition, stimulation of HSG cells with 10 ng/mL IFN-β induced the expression of MX1 (p<0.0001) (**Figure 4E**), IFIT1 (p<0.0001) (**Figure 4F**), IFI44 (p<0.0001) (**Figure 4G**), and IFI44L (p<0.0001) (**Figure 4H**) transcripts. A significant decrease of hsa-miR-145-5p levels were observed in HSG cells stimulated with 10 ng/mL IFN-α (p<0.0001) (**Figure 5A**). IFN-α stimulation also induces the expression of MUC1 (p<0.0001) (**Figure 5B**) and TLR4 (p=0.0012) (**Figure 5C**) transcript levels, which inversely correlated with hsa-miR-145-5p levels (**Figures 5D, E**). Stimulation with 10 ng/mL IFN-β also reduced hsa-miR-145-5p levels (p<0.0001) (**Figure 5F**), and increased MUC1 (p<0.0001) (**Figure 5G**) and TLR4 (p<0.0001) (**Figure 5H**) transcript levels. MUC1 and TLR4 mRNA levels negatively correlated with hsa-miR-145-5p levels in IFN-β stimulated HSG cells (**Figures 5I, J**). In addition, MUC1 and TLR4 protein levels were significantly increased after 10 ng/mL IFN-α or IFN-β stimulation (**Figures 6A–D**).

### hsa-miR-145-5p Overexpression Downregulates MUC1 and TLR4

**Figure 7:**
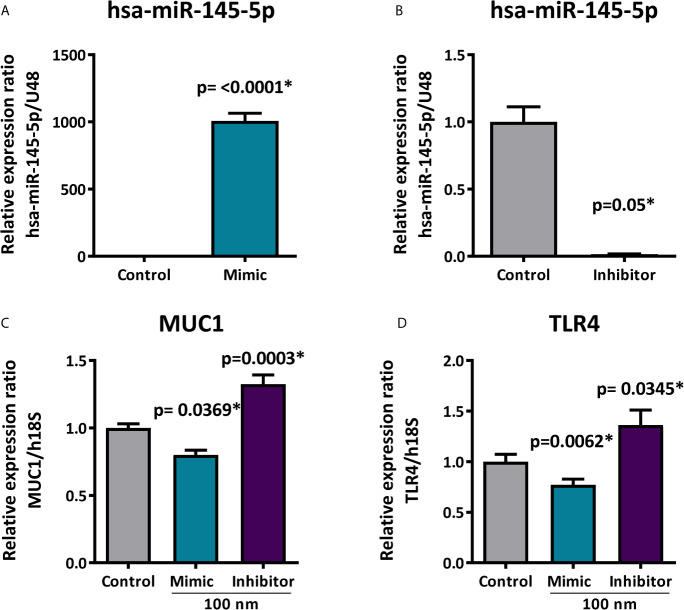
Expression of MUC1 and TLR4 after hsa-miR-145-5p overexpression or inhibition. **(A)** Increased hsa-miR-145-5p levels in HSG cells transfected with the mimic miRNA. **(B)** Decreased hsa-miR-145-5p levels in HSG cells transfected with inhibitor miRNA. **(C)** Relative expression ratio of MUC1 transcripts in HSG cells transfected with mimic hsa-miR-145-5p or inhibitor **(D)** Relative expression ratio of TLR4 transcripts in HSG cells transfected with mimic hsa-miR-145-5p or inhibitor. Data are representative of at least three independent experiments. (*) p-value ≤ 0.05 was considered significant.

Upregulation and downregulation assays identify target genes regulated by specific miRNAs. When evaluating whether hsa-miR-145-5p modulates MUC1 and TLR4 expression in HSG cells, we used mimic and inhibitor miRNAs which are chemically synthesized double-stranded RNA molecules imitating mature miRNA duplexes. HSG cells transfected with 100 nM of mimic hsa-miR-145-5p significantly increased miRNA levels (p<0.0001) (**Figure 7A**), while 100 nM of the hsa-miR-145-5p inhibitor decreased miRNA levels (p=0.05) (**Figure 7B**). MUC1 transcript levels were decreased in HSG cells transfected with mimic hsa-miR-145-5p (p= 0.0369) and increased in HSG cells transfected with the miRNA inhibitor (p=0.0003) (**Figure 7C**). Transfection with the mimic hsa-miR-145-5p significantly decreased TLR4 transcript levels (p=0.0062), and transfection with the miRNA inhibitor increased them (p=0.0345) (**Figure 7D**).

## Discussion

This study aimed to evaluate if miR-145-5p modulates MUC1 and TLR4 expression in LSG from SS-patients in a type I IFNs dependent manner. To our knowledge, this is the first study validating hsa-miR-145-5p levels in LSG from SS-patients by real-time PCR. Our results confirm the observations from a previous study showing a significant decrease of hsa-miR-145-5p in a miRNA expression profile generated by miRNA microarrays in LSG from SS-patients compared with control subjects (29). We observed a negative correlation between hsa-miR-145-5p and type I IFN score in LSG together with our *in vitro* results showing decreased hsa-miR-145-5p levels after IFN-α or IFN-β stimulation. These results suggest that type I IFNs and the activation of its pathway could downregulate hsa-miR-145-5p in LSG from SS-patients. Hsa-miR-145-5p is downregulated in LPS-stimulated macrophages, which depends on type I IFN production and the downstream IFNAR-JAK1-STAT1 signal cascade (28). Functional analyses in LPS-triggered macrophages have demonstrated that hsa-miR-145-5p functions as an anti-inflammatory molecule, promoting IL-10 expression by directly targeting the epigenetic Il10 gene silencer HDAC11 (histone deacetylase 11). In these cells, type I IFNs decrease hsa-miR-145-5p expression, thus alleviating HDAC11 repression, resulting in IL-10 production and precise coordination of innate immune responses (28). Therefore, hsa-miR-145-5p could help avoid overactivation of immune responses and subsequent inflammatory damage to host tissue (28).

In this study, hsa-miR-145-5p inversely correlated with MUC1 and TLR4 mRNA levels, two targets overexpressed in LSG from SS-patients (37–40). Our functional results show reduced MUC1 mRNA levels after transfection with mimic hsa-miR-145-5p, while MUC1 mRNA levels increased after hsa-miR-145-5p inhibition. These results complement those from a previous report showing that MUC1 is a direct target for hsa-miR-145, and suppression of MUC1 is dependent on the 3′-UTR in metastatic breast cancer cell lines, demonstrated by luciferase reporter assays (65). MUC1 has several splicing variants that share the same 3′-UTR: therefore, hsa-miR-145-5p is expected to suppress all variants. This was supported by Western blot results showing decreased levels of large and small isoforms of MUC1 after expression of hsa-miR-145-5p in breast cancer cell lines (65). Hsa-miR-145-5p was downregulated in freshly frozen ovarian carcinoma samples and ovarian carcinoma cell line (SKOV3) based on Northern blot and microarray analysis (66). MUC1 was overexpressed in ovarian (66), breast, colon, pancreas, and bladder tumors and was often associated with the epithelial-mesenchymal transition of different cancer cells, being considered an important metastasis gene (67, 68). Hsa-miR-145-5p overexpression inhibits SKOV3 cell migration and invasion and remarkably reduced the protein but not mRNA expression of MUC1 (69). To further explore the mechanism by which hsa-miR-145-5p inhibits invasion and metastasis, Wang et al. cloned the wild type and mutant miR-145 target sequences of the MUC1 3-’UTR into luciferase reporter vectors and performed the luciferase reporter gene assay, demonstrating that hsa-miR-145-5p negatively regulates MUC1 expression by directly targeting the MUC1 3-’UTR (69). Moreover, MUC1 overexpression alleviated the hsa-miR-145-5p-mediated inhibition of cell invasion, suggesting that miR-145 regulates cell growth and invasion, functioning as a tumor suppressor by directly targeting MUC1 (66). Interestingly, several MUC1 variants are overexpressed and aberrantly localized in LSG from SS-patients (37, 38). Under physiological conditions, salivary mucins are only secreted towards the mouth and are efficient mucosal moisturizers that prevent desiccation (70). One serious problem that SS-patients suffer daily is the dryness of their oral mucosa. We previously demonstrated that this dryness is not just a consequence of the reduced saliva volume (70, 71), but also that mucins play a leading role in mucosa hydration and protection, with hydrophilic groups essentially retaining water molecules in the epithelial surface (70). Previous evidence from our laboratory revealed that MUC1 accumulates in the ER, co-localizing and co-precipitating with GRP78, a chaperone that binds to unfolded proteins in the ER lumen in LSG from SS-patients (38). Also, changes indicative of ER stress and altered unfolded protein response in LSG from SS-patients, such as ER cistern dilation (72), decreased IRE1α/XBP1 pathway activation (73), and ATF6α pathway activation promoting increased ER-associated protein degradation (ERAD) (63) have been observed. In the present study, increased MUC1 mRNA induced by IFN-α and IFN-β together with functional assays using mimic hsa-miR-145-5p and inhibitors suggest that the type I IFN signaling increases MUC1 synthesis mediated by hsa-miR-145-5p inhibition in LSG of SS-patients. This mechanism could contribute to the MUC1 overexpression leading to ER synthesis machinery overload, MUC1 accumulation, and ER stress observed in LSG from SS-patients. MUC1 also induces pro-inflammatory cytokines (46, 49, 50), therefore, MUC1 overexpression mediated through type I dependent hsa-miR-145-5p downregulation could contribute to perpetuating glandular inflammation in LSG from SS-patients.

LSG from SS-patients present significantly increased TLR4 expression compared to controls (39, 40). In the present study, we confirmed TLR4 overexpression in LSG from a different cohort of SS-patients. Also, TLR4 mRNA levels significantly increased after IFN-α and IFN-β stimulation and were repressed by hsa-miR-145-5p overexpression. Hsa-miR-145-5p was down-regulated in high glucose-treated retinal endothelial cells (a cellular model of diabetic retinopathy), and hsa-miR-145-5p overexpression significantly reduced TLR4 at both the protein and mRNA levels (74). Additionally, several studies using luciferase reporter assays provided evidence that the 3’-UTR of TLR4 mRNA is a direct target of hsa-miR-145-5p (74–77). High glucose levels increased TNF-α and IL-1β expression in retinal endothelial cells and were substantially suppressed by hsa-miR-145-5p overexpression, with consequent TLR4 downregulation (74). Furthermore, hsa-miR-145-5p overexpression attenuated the oxidative stress and inflammation induced by high glucose levels, suggesting that hsa-miR-145-5p might exert both anti-oxidative and anti-inflammatory roles in diabetic retinopathy (74). TLR4 expression was decreased in osteoporotic samples compared with nonosteoporotic samples, while hsa-miR-145-5p levels were higher in osteoporotic samples, revealing a significantly negative correlation (75). The luciferase activity of HEK293T cells cotransfected with mimic hsa-miR-145-5p and TLR4‐WT was significantly reduced, while the luciferase activity of cells cotransfected with hsa-miR-145-5p and TLR4‐mutant was similar to control conditions (75). In other study, hsa-miR-145-5p was significantly down-regulated both in mice with acute lung injury and LPS-induced type II alveolar epithelial cells (77). Hsa-miR-145-5p overexpression decreased IL-1β, IL-6, and TNF-α expression levels. Also, it blocked LPS-induced activation of nuclear factor kappa B (NF-κB) pathway and reactive oxygen species (ROS) accumulation in LPS-induced type II alveolar epithelial cells. In these cells, it was confirmed that TLR4 mRNA is a direct target of hsa-miR-145-5p by dual luciferase assays (77). Additionally, hsa-miR-145-5p overexpression alleviated lung tissue injury, decreased the expression levels of IL-1β, IL-6, and TNF-α and reduced myeloperoxidase activity in acute lung injury mouse model, demonstrating that miR-145-5p participated in the progression and development of acute lung injury by decreasing the production of pro-inflammatory cytokines (77). Also, hsa-miR-145-5p inhibits tumor occurrence and metastasis through the NF‐κB signaling pathway by targeting TLR4 in malignant melanoma (76). This miRNA was downregulated in melanoma tissues and cells and suppressed TLR4 expression by binding to its 3’-UTR in melanoma cells (76). Moreover, TLR4 overexpression abolished the inhibition of cell proliferation, colony formation, migration, and invasion abilities induced by hsa-miR-145-5p in melanoma cells (76). Meanwhile, hsa-miR-145-5p restrained melanoma tumor growth *in vivo* by targeting TLR4. Furthermore, hsa-miR-145-5p overexpression inactivated the NF-κB pathway in melanoma *in vitro* and *in vivo*, which was reversed by TLR4 overexpression, suggesting that miR-145-5p hindered the occurrence and metastasis of melanoma cells (76). Also, alterations in cell polarity lead to loss of the innate epithelial barrier function in LSG from SS-patients, triggering a series of changes that result in mucin release to the extracellular matrix (78, 79). Epithelial cell TLR4 recognizes ectopic mucins (mucin obtained from bovine submaxillary glands; MUC5B isolated from human whole saliva; and synthetic sulpho-Lewis (SO_3_-3Galβ1-3GlcNAc) antigen, and initiates a pro-inflammatory response through transcription of IFN-α, IFN-β, TNF-α, IL-6, IL-1β, and CXCL8 in HSG cells (40). These signals, produced initially by epithelial cells, could attract inflammatory cells, perpetuating inflammation and developing a chronic disease (40).

Finally, TLR4 is an N-glycoprotein that is synthesized in the ER and consequently can cause ER stress. The crosstalk between TLR4-mediated signaling and ER stress promotes the production of pro-inflammatory cytokines (80). TLR4 activation can trigger the IRE1α/XBP-1s axis of the UPR and promote pro-inflammatory cytokine expression, such as IL-6 (81). Moreover, TLR4 activation and ER stress inducers synergize the production of pro-inflammatory cytokines, mainly mediated by upregulated p38 expression *via* ATF6 (82): a UPR transcriptional factor increased in SG from SS-patients (63). Since SS-patients exhibit ER stress and ER stress can synergize pro-inflammatory cytokine production mediated by TLR4, overexpression of TLR4 could further favor an inflammatory response in the SG of SS-patients.

In conclusion, our findings suggest that type I IFNs could downregulate hsa-miR-145-5p leading to MUC1 and TLR4 overexpression in LSG from SS-patients. Both MUC1 and TLR4 contribute to inflammation and glandular dysfunction in LSG from SS-patients; thus, these results suggest the anti-inflammatory role of hsa-miR-145-5p and unveil the contribution of type I IFNs in the perpetuation of inflammation.

## Data Availability Statement

The raw data supporting the conclusions of this article will be made available by the authors, without undue reservation.

## Ethics Statement

The studies involving human participants were reviewed and approved by the Ethics Committee of the Facultad de Medicina, Universidad de Chile. The patients/participants provided their written informed consent to participate in this study.

## Author Contributions

DJ, PC, and M-JG conceived the study. DJ and PC conceived and designed the experiments while DJ, PC, IC, M-JB, SA, SG, CM, and MH performed them. SA, SG, and CM were involved in clinical data collection. IC, DJ, PC, M-JB, SA, SG, CM, MH, and MJG wrote the manuscript. All authors contributed to the article and approved the submitted version.

## Funding

This work was supported by Fondecyt-Chile [1210055 to M-JG, SA, IC, CM, SG, M-JB); Fondecyt-Chile [1160015 to M-JG, SA, IC, CM, SG); Enlace-VID Universidad de Chile [ENL04/20 to MJG]; Fondecyt-Iniciación [11170049 to IC]; Fondecyt-Iniciación [11201058 to MJB], and PhD fellowship Conicyt-Chile to DJ and PC.

## Conflict of Interest

The authors declare that the research was conducted in the absence of any commercial or financial relationships that could be construed as a potential conflict of interest.
