# Supplementary material for: Type I Interferon Dependent hsa-miR-145-5p Downregulation Modulates MUC1 and TLR4 Overexpression in Salivary Glands From Sjögren’s Syndrome Patients
Source: Front Immunol. 2021 Jun 2;12:685837. doi: 10.3389/fimmu.2021.685837 (PMC8208490; doi:10.3389/fimmu.2021.685837)
Supplement: Supplementary file 1 [file Table_1.docx]

**Supplementary Table 1.** Sequences of primers used for real time-PCR assays

| **Gene** | **Accession number** | **Primer sequences** |
| --- | --- | --- |
| **MUC1** | NM_001204290.1 NM_001204294.1 NM_001044393.2 NM_001044390.2 NM_001044391.2 NM_001018017.2 NM_001204293.1 NM_002456.5 NM_001204285.1 NM_001204289.1 NM_001204291.1 NM_001204292.1 NM_001204296.1 NM_001044392.2 NM_001204295.1 NM_001204288.1 NM_001018016.2 NM_001204297.1 NM_001204287.1 NM_001204286.1 | F: 5’-GCCACTTCTGCCAACTTGTA-3’  R: 5’-TGAGCTTCCACACACTGAGA-3’ |
| **TLR4** | NM_003266.4 NM_138554.5  NM_138557.3 | F: 5’-TTATTCCCGGTGTGGCCATT-3’  R: 5’-AGCACGACTCGTCAGAAACT-3’ |
| **IFN-α** | NM_024013.3 NM_002172.3  NM_000605.4 NM_006900.4  NM_021068.3 NM_002171.2  NM_002169.3 NM_002173.3  NM_021002.2 NM_002170.4  NM_021057.2 NM_021268.2  NM_002175.2 | F: 5’- AGAGGACCATGCTGACTGATCCATT-3’  R: 5’- CACTGTGCAAAGGTGCACATGAC-3’ |
| **IFN-β** | NM_002176.4 | F: 5’-TCTAGCACTGGCTGGAATGAGACT-3’  R: 5’-TGGCCTTCAGGTAATGCAGAATCC-3’ |
| **MX1** | NM_001144925.2 NM_001178046.3  NM_001282920.1 NM_002462.5 | F: 5’- ACATCCAGAGGCAGGAGACAATCA-3’  R: 5’- CACGTCCACAACCTTGTCTTCAGT-3’ |
| **IFIT1** | NM_001270927.2 NM_001270928.2  NM_001270929.2 NM_001270930.2  NM_001548.5 | F: 5’-AAGAGCCTGCTTTTGGTTGCTG-3’  R: 5’- AGACGGTTGGTTTTGCCATTGC-3’ |
| **IFI44** | NM_006417.5 | F: 5’-AAGGGGTCATTGAGCTCAGGAAGA-3’  R: 5’- TGATGCGTTACATGCCCTTGGA-3’ |
| **IFI44L** | NM_001375646.1 NM_001375648.1  NM_001375647.1 NM_001375649.1  NM_001375650.1 NM_006820.4 | F: 5’- AGCGTTACAGCCCTGCATTTGA-3’  R: 5’- GACTGTTCCAAGGACAGAAGGCAA-3’ |
| **h18S** | NM_022551.2 | F: 5’‐GATATGCTCATGTGGTGTTG‐3’  R: 5’‐AATCTTCTTCAGTCGCTCCA‐3’ |

**Supplementary Table 2.** Antibodies used for Western blot assays

| **Antibody** | **Host species** | **Immunogen** | **Origin** | **Incubation**  **Dilution, time, t°** |
| --- | --- | --- | --- | --- |
| Anti-MUC1, monoclonal (M8), IgG1 | mouse | DTR epitope of MUC1 VNTR | Dr. Dallas Swallow | WB: 1:1000, 20 h, 4°C |
| Anti-TLR4 | rabbit | KLH conjugated synthetic peptide derived from rat TLR4 | Bioss | WB: 1:1000 20 h, 4°C |
| Anti-β-actin, monoclonal (clon BA3R) | mouse | Beta-actin N-terminal peptide. | Invitrogen | WB: 1:10.000, 1 h, RT |
| **Secondary antibodies** | **Company** | | **Host species** | **Dilution** |
| Anti-Rabbit IgG (H+L) Cross-Adsorbed, HRP | Pierce® by Thermo Scientific, 31462 | | Goat | WB: 1:10.000 |
| Anti-Mouse IgG (H+L) Cross-Adsorbed, HRP | Pierce® by Thermo Scientific, 31432 | | Goat | WB: 1:10.000 |
